# Maternal and infant *NR3C1* and *SLC6A4* epigenetic signatures of the COVID-19 pandemic lockdown: when timing matters

**DOI:** 10.1038/s41398-022-02160-0

**Published:** 2022-09-16

**Authors:** Sarah Nazzari, Serena Grumi, Fabiana Mambretti, Marco Villa, Roberto Giorda, Livio Provenzi, MOM-COPE Study Group, Renato Borgatti, Giacomo Biasucci, Lidia Decembrino, Roberta Giacchero, Maria Luisa Magnani, Renata Nacinovich, Federico Prefumo, Arsenio Spinillo, Pierangelo Veggiotti

**Affiliations:** 1 Department of Brain and Behavioral Sciences, University of Pavia, Pavia, Italy; 2 Developmental Psychobiology Lab, IRCCS Mondino Foundation, Pavia, Italy; 3 Molecular Biology Lab, Scientific Institute IRCCS E. Medea, Bosisio Parini, Lecco, Italy; 4 Pediatric Neurosciences Center, IRCCS Mondino Foundation, Pavia, Italy; 5 Department of Pediatrics & Neonatology, Guglielmo da Saliceto Hospital, Piacenza, Italy; 6 Unità Operativa di Pediatria e Nido, ASST Pavia, Vigevano, Italy; 7 Ospedale Civile di Lodi, Lodi, Italy; 8 Unità Operativa di Pediatria e Nido, ASST Pavia, Voghera, Italy; 9 Child and Adolescent Mental Health, San Gerardo Hospital, Monza, Italy; 10 School of Medicine and Surgery & Milan Center for Neuroscience, Università Bicocca, Milan, Italy; 11 Unit of Child and Adolescence Neuropsychiatry, ASST Spedali Civili, Brescia, Italy; 12 Department of Clinical and Experimental Sciences, University of Brescia, Brescia, Italy; 13 Department of Obstetrics and Gynecology, Fondazione IRCCS Policlinico San Matteo, Pavia, Italy; 14 Department of Clinical, Surgical, Diagnostic, and Pediatric Sciences, University of Pavia, Pavia, Italy; 15 Pediatric Neurology Unit, Vittore Buzzi Children’s Hospital, Milan, Italy; 16 Department of Biomedical and Clinical Sciences, L. Sacco, University of Milan, Milan, Italy

**Keywords:** Molecular neuroscience, Predictive markers

## Abstract

Stress exposure during pregnancy is critically linked with maternal mental health and child development. The effects might involve altered patterns of DNA methylation in specific stress-related genes (i.e., glucocorticoid receptor gene, *NR3C1*, and serotonin transporter gene, *SLC6A4*) and might be moderated by the gestational timing of stress exposure. In this study, we report on *NR3C1* and *SLC6A4* methylation status in Italian mothers and infants who were exposed to the COVID-19 pandemic lockdown during different trimesters of pregnancy. From May 2020 to February 2021, 283 mother–infant dyads were enrolled at delivery. Within 24 h from delivery, buccal cells were collected to assess *NR3C1* (44 CpG sites) and *SLC6A4* (13 CpG sites) methylation status. Principal component (PC) analyses were used to reduce methylation data dimension to one PC per maternal and infant gene methylation. Mother–infant dyads were split into three groups based on the pregnancy trimester (first, second, third), during which they were exposed to the COVID-19 lockdown. Mothers and infants who were exposed to the lockdown during the first trimester of pregnancy had lower *NR3C1* and *SLC6A4* methylation when compared to counterparts exposed during the second or third trimesters. The effect remained significant after controlling for confounders. Women who were pregnant during the pandemic and their infants might present altered epigenetic biomarkers of stress-related genes. As these epigenetic marks have been previously linked with a heightened risk of maternal psychiatric problems and less-than-optimal child development, mothers and infants should be adequately monitored for psychological health during and after the pandemic.

## Introduction

During the last decades, evidence has accumulated suggesting that environmental exposures to adverse life events might critically alter the developmental trajectories of infants’ development and mental health by contributing to the epigenetic regulation of stress-related genes [1, 2]. Pregnancy is a sensitive period for women’s mental health [3, 4] as well as for the embedding of environmental exposures into the developmental phenotype of infants [5]. Concerning this latter, previous studies showed that timing and severity of the exposure during gestation are likely to influence the nature and degree of these effects [6], providing further support for the notion that fetal brain development is characterized by a sequence of sensitive time windows, during which specific biological structures and systems, usually undergoing rapid developmental change at that given point, are particularly vulnerable to environmental influences. Thus, for example, studies on the risk for schizophrenia indicated that the most sensitive period for prenatal exposure to adversity was the first trimester [7] when the processes of neuronal migration occur. In contrast, the strongest effects of antenatal adverse exposure on cognitive outcomes [8], stress regulation [9, 10, 109] and offspring’s emotional problems [11] have been more frequently reported in late pregnancy when rapid fetal brain development, including synaptogenesis, neural migration, and the beginning of synaptic differentiation, takes place.

Early stressful or traumatic events that occur during the first 1000 days—and especially during pregnancy—might leave stable signatures in the epigenome of mothers and infants [12, 13]. Changes in the DNA methylation status of specific portion of stress-related genes are among the most studied epigenetic signatures of early exposures to adverse life events. DNA methylation is fostered by DNA methyltransferase enzymes (DNMTs) that are responsible for the binding of the methyl group from donor S-adenosyl-methionine onto the 5’ position of the CpG dinucleotide. DNA methylation occurring in regions relevant for gene regulation and expression, characterize by high density of CpG sites (i.e., CpG islands) are of great concern for researchers and clinicians. These regions include exons, promoter regions, and enhancers. Nonetheless, methylation occurring in other regions is thought to interact with other epigenetic mechanisms and to contribute to the emergence of increased or reduced transcriptional sensitivity. For instance, higher methylation in both exonic and intronic regions is effective in recruiting histone deacetylases, leading to gene silencing. DNA methylation usually alter the accessibility of a gene coding region to the molecular transcriptional agents and could lead to altered expression and a consequently altered availability of proteins that are necessary for the adequate development of individuals. The methylation dynamics of genes encoding for the glucocorticoid receptor (*NR3C1*) and the serotonin transporter (*SLC6A4*) are key targets of behavioral epigenetics, as they are deeply involved in stress regulation and mental health.

The hypothalamic–pituitary–adrenal (HPA) axis is key in regulating the mobilization of energy in the organism [14]. It is involved in stress reactivity and regulation and it supports the development of behavioral, cognitive, and socio-emotional domains as well as mental health. The HPA axis response to challenging and stressful conditions involves a cascade of hormonal activations that finally leads to secreting glucocorticoids (cortisol in humans) from the adrenal glands. Cortisol secretion is regulated by feedback mechanisms that involve the activation and binding of the hormone to specific glucocorticoid receptors (GRs) in the brain. The *NR3C1* gene encodes for specific GRs in the mammalian brain and is epigenetically regulated by environmental exposures. The epigenetic regulation of the *NR3C1* gene is highly sensitive to environmental adverse and protective conditions during sensitive periods, including pregnancy. Available evidence shows that the methylation status of NR3C1 gene in cord blood was predicted by maternal psychological distress [15–17], experiences of war-related stress [18, 19] and partner violence [20] during pregnancy.

The serotonergic neurotransmission is also known to affect a wide range of developmental outcomes in infants and mental health in adults. In humans, serotonin is located both in the central nervous system and in peripheral tissues [21]. In the brain, it is located in the neurons of the median and dorsal raphe nuclei, in the cortex, and in the hippocampus. The amygdala, hypothalamus, and the pituitary adrenal gland, which are deeply involved in stress regulation mechanisms, are densely innervated by serotonin neurons [22]. The serotonin transporter is a crucial regulator of the serotonergic system: it removes the serotonin released in the synaptic cleft, and it is synthesized by a specific gene, namely the *SLC6A4*. The expression of this gene is regulated by polymorphic allelic variants and epigenetic mechanisms [23, 24]. A systematic review has recently reported on the potential of the *SLC6A4* gene’s methylation as a biomarker of exposure to life adversity which also associates with less-than-optimal outcomes in child development and adult mental health [12]. However, few studies have investigated the link between antenatal exposure to adversity and *SCL6A4* gene methylation and these have yielded inconsistent findings. Either negative [25], positive [26], or null [27] associations between maternal antenatal psychological distress and infant SLC6A4 methylation patterns have been reported.

The COVID-19 pandemic dramatically hit northern Italy at the beginning of 2020 [28]. With the increasing number of patients requiring intensive care and with unprecedented mortality rates associated with this clinical condition, the COVID-19 pandemic rapidly grew as a potential collective trauma. The effects of pandemic-related stress have been already highlighted in healthcare professionals working at the forefront of the pandemic emergency [29, 30] as well as in the general population [31]. Moreover, individuals who are experiencing periods of heightened neuroplasticity might be especially sensitive to environmental stress and life adversity. Not surprisingly, a large number of studies are documenting detrimental associations of pandemic-related stress with both infants’ developmental outcomes and maternal mental health indexes. Epigenetic pathways are likely to play a role in mediating the impact of exposure to the pandemic during gestation on maternal and fetal phenotypes, yet they are still largely unknown. It is important to obtain information on the potential epigenetic pathways underlining these associations in order to increase our knowledge of the neurobiology of stress. Furthermore, major stressful events, such as the pandemic or natural disasters, provide the unique opportunity to test for timing effects [32], which are rarely addressed in studies examining the epigenetic underpinnings of antenatal stress exposures. Obtaining information on time periods during pregnancy differentially sensitive to epigenetic regulation by life adversities is key to provide innovative evidence basis to the programming of preventive and care actions during and after the pandemic time.

In this study, we report on *NR3C1* and *SLC6A4* methylation in mothers and infants who were exposed to the initial stage of COVID-19 pandemic in Northern Italy—the first lockdown period—between March and May 2020. We focused on the first lockdown period as this constituted an acute major stressful event for the Italian population with a clear and sudden onset and characterized by features of uncertainty, fear, and lack of social support [33]. This provides greater leverage for exploring timing effects of pandemic stress-related exposure across gestation on maternal and infant epigenome. More specifically, we assessed the presence of significant differences in *NR3C1* and *SLC6A4* methylations among those individuals—both mothers and infants—who experienced the lockdown period during different trimesters of pregnancy.

## Methods

### Participants

The Measuring the Outcomes of Maternal COVID-19-related Prenatal Exposure (MOM-COPE) research project is a longitudinal and multi-centric cohort study that involves ten neonatal units in Northern Italy [34]. The study is registered at ClinicalTrials.gov with unique identifier NCT04540029. Here, we report cross-sectional evidence on a sample of 283 mother–infant dyads who provided complete self-report and biological data at delivery. The dyads were enrolled from May 2020 to February 2021. Mothers were included if at least 18 years old, in the absence of prenatal and perinatal diseases or injuries, if they delivered at term (i.e., from 37 + 0 to 41 + 6 weeks of gestational age), and if they were negative for SARS-CoV-2 at delivery as by PCR testing. Mothers were first contacted at antepartum classes or immediately following the postpartum period. Socio-demographic and neonatal data were obtained from medical records. The study was approved by the Ethics Committees of the project lead institution (IRCCS Mondino Foundation, Pavia, Italy) and the participating hospitals. All the procedures were performed in accordance with the 2018 Declaration of Helsinki for studies conducted with human participants. All mothers provided informed consent to participate to the study.

### Context

To further characterize the present sample and to optimize the possibility of cross-country comparisons, we report a brief description of the historical context of COVID-19 pandemic in the local area where the study was conducted. The neonatal units were all placed in the Northern Italy region of the first SARS-CoV-2 spread – specifically among the provinces of Bergamo, Cremona, Lodi, Pavia, and Piacenza. The first Italian COVID-19 case in this region was officially registered on February 21, and the first death on February 22. By the first week of March, the virus had spread to multiple regions in the Italian territory. The first lockdown was issued on March 8 and was in place until June 3.

### Procedures and measures

Within 48 h from delivery, mothers self-reported on depressive symptoms and anxiety by replying to an online adapted version of the well-validated Beck Depression Inventory (BDI-II [35] and State-Trait Anxiety Inventory (STAI-Y [36]) questionnaires, respectively. The Italian version of the BDI-II [37] is a 21-item self-report questionnaire that provides a descriptive and non-diagnostic account of the severity of symptoms of depression. Each item is rated on a 4-point Likert scale, and the total continuous score ranges from 0 (low) to 63 (high). The state anxiety subscale of the Italian version of the STAI-Y [38] was used here; it features 20 4-point Likert-scale items and provides a descriptive and non-diagnostic account of the severity of symptoms of anxiety. The total continuous score ranges from 20 (low) to 80 (high).

Between 6 and 24 h from delivery, buccal cells were obtained from mothers and infants using OraCollect for Pediatrics kit OC-175 (DNA Genotek, Ottawa, Canada). Methylation assessment was conducted according to previous validated procedures from this lab [26, 39]. The genomic DNA was extracted following the manufacturer’s protocols, and its quality was assessed using a Qubit fluorometer Invitrogen, Thermo Fisher Scientific, Waltham, Massachusetts, USA. The methylation status of the SLC6A4 gene’s region (chr17:28562750-28562958; 13 CpGs) and NR3C1 gene’s region (chr5:142763694-142764254; 44 CpG sites) was assessed by PCR amplification of bisulfite-treated DNA followed by next-generation sequencing (NGS) on a NEXTSeq-500 (Illumina, San Diego, California, USA). Target regions were chosen based on previous literature on the serotonin transporter gene and glucocorticoid receptor gene epigenetic regulation by stress exposure [12, 40]. The position of each CpG site is reported in Supplementary File S1.

### Data reduction

The trimester of exposure to the lockdown was obtained by considering the actual dates of lockdown in Italy (see “Context”) and according to the date of birth of the included infants. Consequently, infants born between May and July had been exposed to the lockdown during the third trimester, those born between August and October had been exposed during the second trimester, and the remaining infants born after October have been assigned to the first-trimester group. Among the 283 dyads, three mothers had no valid *NR3C1* methylation data and were thus excluded from the present sample leaving 280 available mothers for the analyses. For infant *NR3C1* and for both maternal and infants *SLC6A4* methylation, complete data were available. The reduce the number of testable CpG sites on maternal and infant *SLC6A4* and *NR3C1*, separate principal component analyses were carried setting simplimax rotation [41], suppressing coefficients lower than 0.40, and extracting principal components (PCs) based on eigenvalue greater than one (Revelle, 2019). For mothers, 12 out of 13 *SLC6A4* CpG sites loaded on one unique PC, M-*SLC6A4*, which explained 51% of variance in maternal *SLC6A4* methylation, whereas 15 out of 44 CpG *NR3C1* CpG sites loaded on a PC, M-*NR3C1*, which explained 14% of total variance in maternal *NR3C1* methylation. For infants, 10 out of 13 *SLC6A4* CpG sites loaded on one PC, I-*SLC6A4*, which explained 29% of the variance in infant *SLC6A4* methylation, whereas 16 out of 44 CpG *NR3C1* CpG sites loaded on a PC, I-*NR3C1*, which explained 14% of total variance in infant *NR3C1* methylation. The results of the four principal component analyses are reported in details in Supplementary File S2. These four PCs were included in further analyses.

### Plan of analyses

First, the presence of significant differences in socio-demographic, neonatal, and maternal mental health variables among mother–infant dyads who were exposed to the COVID-19 pandemic lockdown during the third, second, or first trimester of pregnancy was explored by means of one-way analyses of variance (ANOVAs). To assess the presence of significant differences in maternal and infant methylation of *SLC6A4* and *NR3C1* genes by pregnancy trimester of exposure to the COVID-19 pandemic lockdown, four separate one-way ANOVAs were carried out with Trimester (levels: third, second, first) as the between-subject variable and each of the methylation PCs. Significant ANOVA effects were further explored by means of Tukey-adjusted post hoc tests. A correlation matrix was used to identify potential confounding associations among socio-demographic, neonatal, and maternal mental health variables and maternal and infant methylation of the *SLC6A4* and *NR3C1* genes. Variables for which a significant association emerged were included together with Trimester in a general linear model (GLM) [42] to estimate variations in *SLC6A4* and *NR3C1* genes’ methylation; models were run separately for each gene as well as mothers and infants. All the analyses were performed with JAMOVI 2.2.5 [43] and setting *P* < 0.05.

## Results

**Fig. 1:**
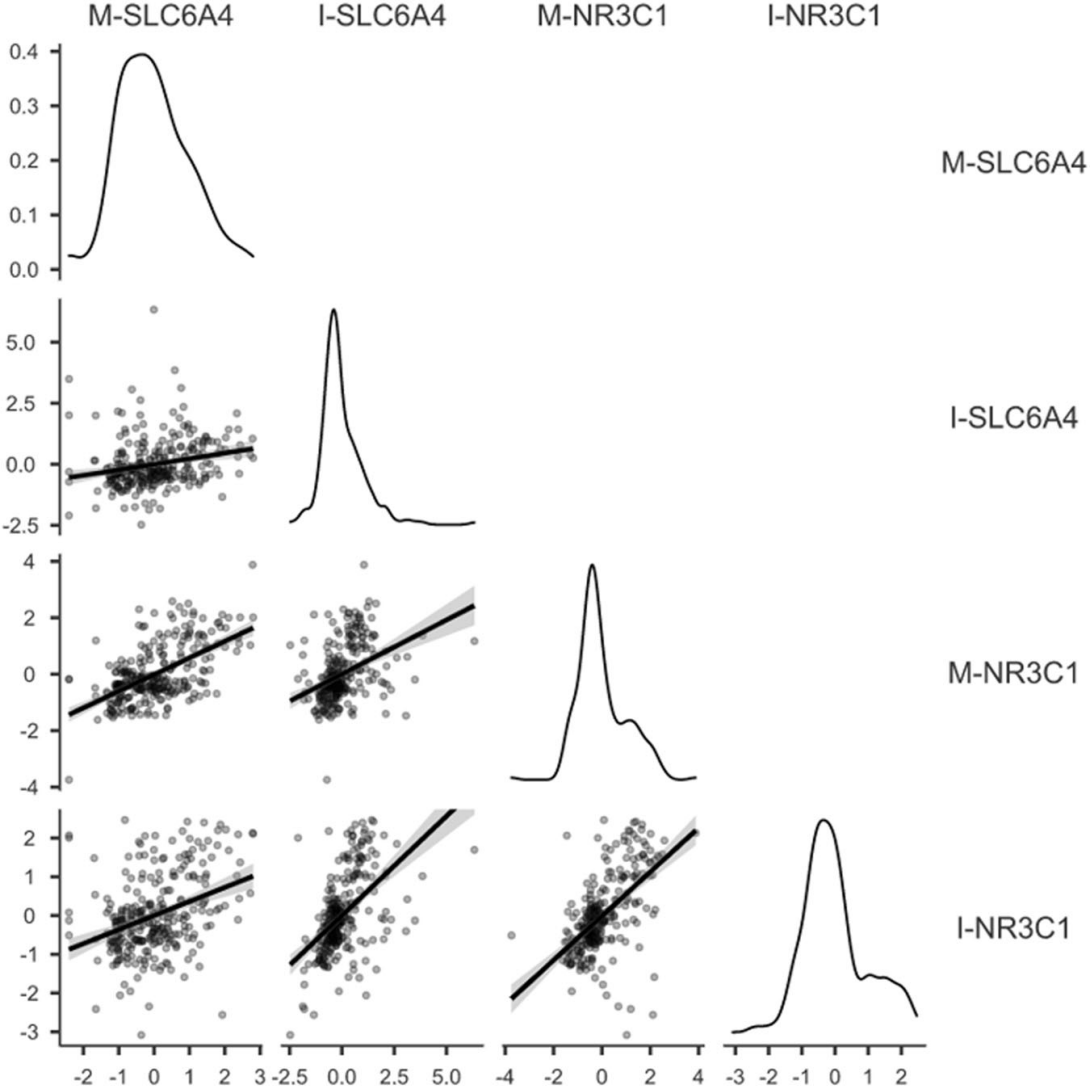
Correlation matrix for maternal and infant *SLC6A4* and *NR3C1* principal component scores. *X*- and *Y* axes report methylation percentage. Density plots on diagonal. Note. ***P < 0.001.

Socio-demographic and neonatal variables are reported in Table 1. No significant differences by Trimester emerged for infants’ gestational age, birth weight, head circumference, and Apgar score at minute 1 and minute 5. No differences by Trimester emerged for maternal anxiety and depression. At post hoc, mothers who were exposed to the COVID-19 pandemic lockdown during the first trimester were older (mean age = 34.43 years, SD = 3.64) than counterparts exposed during the second (mean age = 32.81, SD = 4.30) and third trimester (mean age = 32.80 years, SD = 4.85) of pregnancy. No statistically significant differences emerged by sex in I-*SLC6A4*, *t*(281) = 0.16, *P* = 0.875, and I-*NR3C1*, *t*(281) = 0.80, *P* = 0.423, methylation scores. The correlations between maternal and infant *SLC6A4* and *NR3C1* methylation PC scores are reported in Fig. 1. As such maternal age was included as a covariate in the following ANOVAs on methylation PCs. Maternal depression and anxiety scores were not significantly correlated with infants and maternal methylation PC scores.

**Table 1:** Sample characteristics.

	Whole sample *N* = 283		Third trimester *N* = 118		Second trimester *N* = 84		First trimester *N* = 81		Comparison	
	Mean	SD	Mean	SD	Mean	SD	Mean	SD	*F*	
Gestational age (weeks)	39.66	1.08	39.76	1.02	39.75	1.11	39.42	1.09	3.04	ns
Birth weight (grams)	3347.42	433.49	3376.44	435.89	3331.85	397.14	3321.30	467.65	0.46	ns
Head circumference (cm)	34.20	1.21	34.41	1.29	34.09	0.99	34.18	1.27	1.97	ns
Apgar at 1 min	9.20	0.69	9.26	.59	9.15	0.65	9.15	.85	0.46	ns
Apgar at 5 min	9.87	0.37	9.92	.30	9.83	0.43	9.84	.37	0.14	ns
Maternal age (years)	33.27	4.42	32.80	4.85	32.81	4.30	34.42	3.64	3.94	*
Maternal depression (BDI score)	7.29	5.86	6.07	5.45	6.82	6.45	7.30	5.16	0.31	ns
Maternal anxiety (STAI score)	34.08	10.60	35.34	9.85	35.35	10.63	37.56	9.55	0.24	ns
	***N***	**%**	***N***	**%**	***N***	**%**	***N***	**%**	***Χ***^2^	
Sex: males	141	49.8	141	49.8	141	49.8	141	49.8	0.16	ns
Sex: females	142	50.2	142	50.2	142	50.2	142	50.2		

Note. ns, *P* ≥ 0.0, *P < 0.05.

**Fig. 2:**
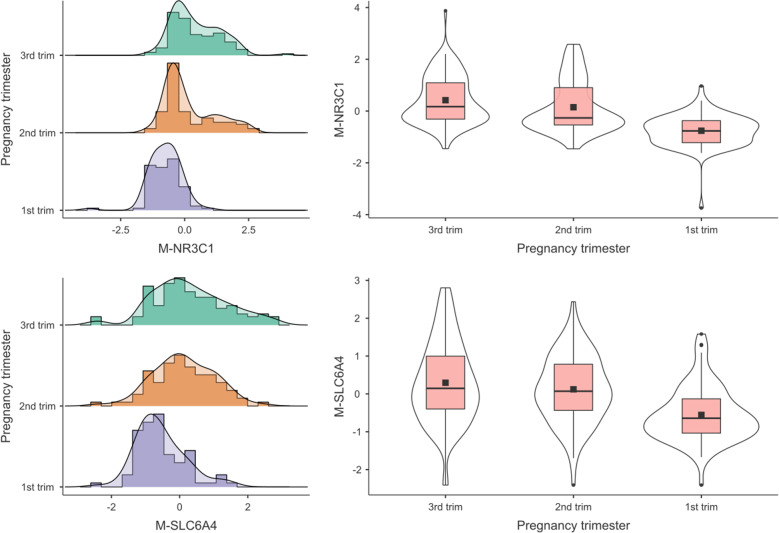
Maternal *SLC6A4* and *NR3C1* methylation by pregnancy trimester. Note. ***P < 0.001.

**Fig. 3:**
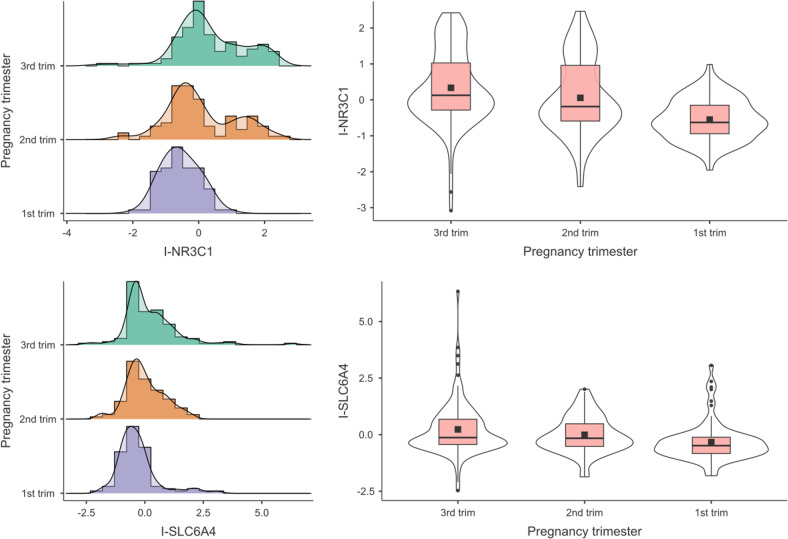
Infants’ *SLC6A4* and *NR3C1* methylation by pregnancy trimester. Note. ***P < 0.001.

Significant differences in DNA methylation by Trimester of exposure to COVID-19 pandemic lockdown emerged for mothers (see Fig. 2), M-*SLC6A4*, *F*(2,280) = 20.79, *P* < 0.001, M-*NR3C1*, *F*(2,277) = 45.73, *P* < 0.001, and for infants (see Fig. 3), I-*SLC6A4*, *F*(2,280) = 7.96, *P* < 0.001, I-*NR3C1*, *F*(2,280) = 21.69, *P* < 0.001. The effects remained significant when including maternal age as a covariate (Supplementary File S3). Post hoc tests revealed that in dyads exposed to the COVID-19 pandemic lockdown during the first trimester of pregnancy the methylation status of both genes was lower compared to counterparts exposed during the second or third trimester (M-*SLC6A4*: 3rd trimester vs. 2nd trimester, *t*(280) = 1.30, *P* = 0.398; 3rd trimester vs. 1st trimester, *t*(280) = 6.29, *P* < 0.001; 2nd trimester vs. 1st trimester, *t*(280) = 4.64, *P* < 0.001; I-*SLC6A4*: 3rd trimester vs. 2nd trimester, *t*(280) = 1.70, *P* = 0.206; 3rd trimester vs. 1st trimester, *t*(280) = 3.99, *P* < 0.001; 2nd trimester vs. 1st trimester, *t*(280) = 2.14, *P* = 0.085; M-*NR3C1*: 3rd trimester vs. 2nd trimester, *t*(277) = 2.17, *P* = 0.079; 3rd trimester vs. 1st trimester, *t*(277) = 9.38, *P* < 0.001; 2nd trimester vs. 1st trimester, *t*(277) = 6.67, *P* < 0.001; I-*NR3C1*: 3rd trimester vs. 2nd trimester, *t*(280) = 2.09, *P* = 0.094; 3rd trimester vs. 1st trimester, *t*(280) = 6.55, *P* < 0.001; 2nd trimester vs. 1st trimester, *t*(280) = 4.16, *P* < 0.001).

## Discussion

In this study, we assessed the presence of a significant difference in the methylation status of target regions of the *SLC6A4* and *NR3C1* genes in mothers and infants who were exposed to the COVID-19 pandemic lockdown during different trimesters of pregnancy. The findings provide novel evidence suggesting the presence of a similar trend for both mothers and infants and for both genes examined, with a heightened sensitivity to epigenetic upregulation by life adversities when the lockdown was experienced during the second and third trimesters of pregnancy compared to the earlier gestation period.

For what pertains to infant *NR3C1* and *SLC6A4* methylation, previous research suggested that prenatal exposure to life adversities may link with altered patterns of methylation, although evidence concerning the direction of the associations is inconclusive [44]. Differences related to the specific trimester of exposure might contribute to findings inconsistency; however, they were not systematically addressed in previous human studies. For instance, Stonawski et al. [45] found a significant positive association between maternal depression during the third trimester of pregnancy and school-age children’ *SLC6A4* and *NR3C1* CpG-specific methylation. Nonetheless, the effect of maternal depression during other trimesters was not investigated. Devlin et al. [25] reported on the association of maternal prenatal depressed mood during the second trimester of pregnancy—but not the third trimester—with lower infant *SLC6A4* promoter methylation at birth. Oberlander and colleagues [15] found a significant association of maternal prenatal depression during the third trimester of pregnancy—but not the second trimester—and methylation of NR3C1 in newborns. Maternal community deprivation during the second or third trimester of pregnancy was found to be significantly associated with greater infant DNA methylation in eight CpG sites of the *SLC6A4* gene, but not of the *NR3C1* gene [46]. Dereix et al. [47] recently reported higher NR3C1 methylation in infants of mothers with high levels of prenatal anxiety, but not depression, although gestational timing of exposure was not specified. Similarly, in a cohort of 83 pregnant women, prenatal maternal anxiety was found to be significantly linked with infant *NR3C1* methylation in specific CpG sites located into exon 1F [48]. Notably, in this study the association between prenatal adversity (i.e., maternal anxiety) and the epigenetic regulation of the glucocorticoid receptor gene was documented for all three trimesters. A recent meta-analysis published by Palma-Gudiel and colleagues [49] was supportive of an association between prenatal exposure to life adversities, and CpG-specific increased in the methylations status of the *NR3C1* gene promoter. Nonetheless, the meta-analytic study was inconclusive on the role of pregnancy timing as a mediator of the association, as in a very limited subset of original papers the specific trimesters were reported separately.

Accordingly, the present findings contribute to cover a gap in the existent literature, as they highlight a unique contribution of adversities occurring during the second and third trimesters of pregnancy—and not the first— on the epigenetic regulation of the *SLC6A4* and *NR3C1* gene in infants. Current findings suggest that those genes are, to some extent, developmentally sensitive to prenatal exposure to a major stressful event such as the pandemic. Questions remain about the mechanisms by which pandemic exposure in late pregnancy might initiate greater epigenetic changes in fetal brain development, as compared to earlier exposures. Biological alterations, for example, in levels of cortisol or inflammatory markers, underline maternal antenatal stress experiences [50, 51] and are likely to affect the transcriptional and epigenetic activity during critical periods of development [1]. Notably, the fetal brain is partially protected from elevations in maternal cortisol by the activity of the placental 11b-hydroxysteroid-dehydrogenase type 2 (11-βHSD2); it has been shown that the expression of this enzyme is reduced in the last stages of gestation, thus allowing more cortisol to cross the placenta and influence fetal brain development [52]. Furthermore, the fetal HPA axis is not fully developed until the second trimester, so it is possible that stress-related effects on this system may not be apparent unless the stressors occur late in gestation [53].

Noteworthy, maternal and fetal physiology are strongly connected across pregnancy [54]. In line with this notion, maternal and infant levels of methylation of the *NR3C1* and *SLC6A4* genes were moderately to strongly associated in the current sample. Further, we provided evidence of an association between timing of prenatal exposure to the COVID-19 lockdown and maternal methylation of the *NR3C1* and *SLC6A4* genes, which parallels the association we reported in newborns. The association between environmental adversity and methylation of the *NR3C1* or *SCL6A4* genes is well-established in the general population [55, 56]. Less is known regarding how stressful experiences influence the maternal epigenome during pregnancy. Literature on the effects of life adversities experienced during pregnancy and maternal *NR3C1* and *SLC6A4* genes’ methylation is limited. Kertes and colleagues [57] found an association between chronic stress and war-related trauma in levels of maternal *NR3C1* gene methylation soon after delivery in a small sample of Congolese women (*N* = 24) exposed to severe war-related stressors during pregnancy. Further, changes in methylation of *NR3C1* gene were reported in women who were exposed to interpersonal violence [58] and to the Tutsi genocide during pregnancy [59]. Devlin and colleagues [25] reported a negative association between maternal depressive symptoms in the second—but not third—trimester of pregnancy and maternal methylation levels in the *SLC6A4* gene. Albeit preliminary, the current findings extend available evidence by showing that the second and third trimester of pregnancy might represent more vulnerable windows for the effects of stressful experiences on maternal epigenetic regulation of genes involved in stress regulation mechanisms. Importantly, levels of maternal depressive or anxiety symptoms were unrelated to methylation of target genes in the current sample, thus possibly suggesting that the objective stress experience, rather than the subjective one, might be more closely implicated in shaping maternal epigenetic regulation. However, this hypothesis needs to be explicitly addressed in future studies. Mechanisms underlying the observed effects of timing of exposure on maternal methylation remain an open area for future inquiry. Maternal stress response systems undergo substantial changes as gestation advances in order to support fetal growth and development. Generally, an increase in cortisol levels and pro-inflammatory cytokines have been reported in the third trimester of pregnancy [60, 61]. We might speculate that a greater than typical increase in stress hormones and inflammatory markers related to the stressful experience of the pandemic in the third trimester of pregnancy might affect more significantly maternal epigenetic regulation, leading to greater methylation of stress-related genes in late gestation.

Interpretation of these findings should be carefully done considering the following limitations. First, *NR3C1* and *SLC6A4* methylation were peripherally assessed in buccal cells. It is unclear how epigenetic variation in the peripheral tissue relates to epigenetic change within the brain. Partial evidence exists on the cross-tissue consistency of DNA methylation measures in humans. It is plausible that the methylation status of genes that have widespread effects and actions across central and peripheral tissues—such as the *NR3C1* and the *SLC6A4* genes—may be tissue- and site-specific [62–64]. Second, previous review [12] and meta-analytic [40] evidence were not conclusive on the directionality of epigenetic regulatory mechanisms in association with adverse exposures. While increased methylation of *SLC6A4* and *NR3C1* genes is reported in many papers, patterns of hypomethylation have been also reported in individuals exposed to adversities [65] or in subjects with less-than-optimal outcomes [66]. From this perspective, our findings do not necessarily suggest the presence of better or worse outcomes according to the trimester of adversity of exposure; rather, they highlight the presence of different gradients of susceptibility, which might be reflected in increased or reduced patterns of methylation in stress-related genes. The functional and adaptive consequences of hyper- and hypomethylation patterns cannot be conclusively addressed here. Third, while we examined target genes related to stress regulation and serotonin transmission, it should be underlined that several different genomic regions might be impacted by the stress-related pandemic exposure and also that gene polymorphisms are likely to moderate the impact of stress and their impact should not be underestimated. Fourth, the lack of a pre-pandemic control group is another limitation of this study. As we cannot compare the present sample with mother–infant dyads who were not exposed to the COVID-19 pandemic, complementary or additional explanations of our findings might be considered. For instance, it is possible that those exposed during the first trimester of pregnancy had more time to recover from the traumatic experience of the lockdown. Similarly, a proximity effect between exposure and testing might further act as an uncontrolled bias in the absence of a non-pandemic control condition. Fifth, while neonatal levels of methylation were measured soon after birth and are, thus, independent of the effects of the postnatal environment, we did not measure additional sources of antenatal stress, whose effects on maternal and fetal methylation levels could not be ruled out. For example, we did not control for the number of previous children or other traumatic experiences that may have occurred in women’s life. Lastly, interpretation of findings should be cautious as findings are correlational, and causality cannot be inferred from this initial study.

## Conclusions

The outbreak of COVID-19 pandemic represents an unprecedented adversity for the global population which might have potentially long-term effects, particularly in vulnerable populations, such as women who were pregnant at the time of the pandemic and their infants. While the literature on the impact of the pandemic on women and infant mental health is burgeoning, little is known about the possible underlining biological mechanisms and windows of vulnerability. The current study is among the first to show a greater impact of exposure to the COVID-19 pandemic on levels of maternal and infant methylation of target stress-related genes (i.e., *NR3C1* and *SLC6A4*) in late gestation, as compared to earlier exposures. It is noteworthy that high methylation of the *NR3C1* gene has been linked with altered stress regulation [15] and poorer neurodevelopmental outcomes [67]. Likewise, greater methylation of the *SCL6A4* gene has been associated with altered socio-emotional development and stress regulation [12, 26]. Future research will help to clarify whether the observed heightened methylation at these sites has functional consequences for maternal and infant health. This knowledge could allow for timely identification of and intervention with high-risk mother–infant dyads.

## Supplementary information

Supplementary File S1

Supplementary File S2

Supplementary File S3

## References

[1] Kundakovic M, Jaric I. The epigenetic link between prenatal adverse environments and neurodevelopmental disorders. Genes. 2017;8. 10.3390/genes8030104. 10.3390/genes8030104 PMC5368708 28335457

[2] Vaiserman AM, Koliada AK (2017). Early-life adversity and long-term neurobehavioral outcomes: epigenome as a bridge?. Hum Genomics.

[3] Biaggi A, Conroy S, Pawlby S, Pariante CM (2016). Identifying the women at risk of antenatal anxiety and depression: a systematic review. J Affect Disord.

[4] Falah-Hassani K, Shiri R, Dennis CL (2017). The prevalence of antenatal and postnatal co-morbid anxiety and depression: a meta-analysis. Psychological Med.

[5] Weinstock M (2008). The long-term behavioural consequences of prenatal stress. Neurosci Biobehav Rev.

[6] Kapoor A, Petropoulos S, Matthews SG (2008). Fetal programming of hypothalamic–pituitary–adrenal (HPA) axis function and behavior by synthetic glucocorticoids. Brain Res Rev.

[7] Khashan AS, Abel KM, McNamee R, Pedersen MG, Webb RT, Baker PN (2008). Higher risk of offspring schizophrenia following antenatal maternal exposure to severe adverse life events. Arch Gen Psychiatry.

[8] Davis EP, Sandman CA (2010). The timing of prenatal exposure to maternal cortisol and psychosocial stress is associated with human infant cognitive development. Neurosci Biobehav Rev.

[9] Davis EP, Glynn LM, Waffarn F, Sandman CA (2011). Prenatal maternal stress programs infant stress regulation. J Child Psychol Psychiatry Allied Discip.

[10] Vedhara K, Metcalfe C, Brant H, Crown A, Northstone K, Dawe K (2012). Maternal mood and neuroendocrine programming: effects of time of exposure and sex. J Neuroendocrinol.

[11] Rice F, Jones I, Thapar A (2007). The impact of gestational stress and prenatal growth on emotional problems in offspring: a review. Acta Psychiatr Scandinavica.

[12] Provenzi L, Giorda R, Beri S, Montirosso R (2016). SLC6A4 methylation as an epigenetic marker of life adversity exposures in humans: a systematic review of literature. Neurosci Biobehav Rev.

[13] Maccari S, Krugers HJ, Morley-Fletcher S, Szyf M, Brunton PJ (2014). The consequences of early-life adversity: neurobiological, behavioural and epigenetic adaptations. J Neuroendocrinol.

[14] Tsigos C, Chrousos GP (2002). Hypothalamic-pituitary-adrenal axis, neuroendocrine factors and stress. J Psychosom Res.

[15] Oberlander TF, Weinberg J, Papsdorf M, Grunau R, Misri S, Devlin AM (2008). Prenatal exposure to maternal depression, neonatal methylation of human glucocorticoid receptor gene (NR3C1) and infant cortisol stress responses. Epigenetics.

[16] Braithwaite E, Kundakovic M, Ramchandani P, Murphy S, Champagne F (2015). Maternal prenatal depressive symptoms predict infant NR3C1 1F and BDNF IV DNA methylation. Epigenetics.

[17] Mansell T, Vuillermin P, Ponsonby A-L, Collier F, Saffery R, Ryan J (2016). Maternal mental well-being during pregnancy and glucocorticoid receptor gene promoter methylation in the neonate. Dev Psychopathol.

[18] Mulligan CM, Friedman JE (2017). Maternal modifiers of the infant gut microbiota: metabolic consequences. J Endocrinol.

[19] Kertes DA, Bhatt SS, Kamin HS, Hughes DA, Rodney NC, Mulligan CJ (2017). BNDF methylation in mothers and newborns is associated with maternal exposure to war trauma. Clin Epigenetics.

[20] Radtke KM, Ruf M, Gunter HM, Dohrmann K, Schauer M, Meyer A (2011). Transgenerational impact of intimate partner violence on methylation in the promoter of the glucocorticoid receptor. Transl Psychiatry.

[21] Charnay Y, Leger L (2010). Brain serotonergic circuitries. Dialogues Clin Neurosci.

[22] Berger M, Gray JA, Roth BL (2009). The expanded biology of serotonin. Annu Rev Med.

[23] Canli T, Lesch K-P (2007). Long story short: the serotonin transporter in emotion regulation and social cognition. Nat Neurosci.

[24] Houwing DJ, Buwalda B, van der Zee EA, de Boer SF, Olivier JDA. The serotonin transporter and early life stress: translational perspectives. Front Cell Neurosci. 2017;11. https://www.frontiersin.org/articles/10.3389/fncel.2017.00117. 10.3389/fncel.2017.00117 PMC5405142 28491024

[25] Devlin AM, Brain U, Austin J, Oberlander TF (2010). Prenatal exposure to maternal depressed mood and the MTHFR C677T variant affect SLC6A4 methylation in infants at birth. PLoS ONE.

[26] Provenzi L, Mambretti F, Villa M, Grumi S, Citterio A, Bertazzoli E (2021). Hidden pandemic: COVID-19-related stress, SLC6A4 methylation, and infants’ temperament at 3 months. Sci Rep..

[27] Dukal H, Frank J, Lang M, Treutlein J, Gilles M, Wolf IA (2015). New-born females show higher stress- and genotype-independent methylation of SLC6A4 than males. Borderline Personal Disord Emot Dysregul.

[28] Spina S, Marrazzo F, Migliari M, Stucchi R, Sforza A, Fumagalli R (2020). The response of Milan’s emergency medical system to the COVID-19 outbreak in Italy. Lancet.

[29] Barello S, Palamenghi L, Graffigna G (2020). Burnout and somatic symptoms among frontline healthcare professionals at the peak of the Italian COVID-19 pandemic. Psychiatry Res.

[30] Gagliardi L, Grumi S, Gentile M, Cacciavellani M, Placidi G, Vaccaro A, et al. The COVID-related mental health load of neonatal healthcare professionals: a multicentre study in Italy. Ital J Pediatr. 2022;8:136. 10.1186/s13052-022-01305-7 PMC9338560 35907872

[31] Fiorillo A, Gorwood P (2020). The consequences of the COVID-19 pandemic on mental health and implications for clinical practice. Eur Psychiatry: J Assoc Eur Psychiatrists.

[32] King S, Dancause K, Turcotte-Tremblay A-M, Veru F, Laplante DP (2012). Using natural disasters to study the effects of prenatal maternal stress on child health and development. Birth Defects Res Part C: Embryo Today: Rev.

[33] Thomason ME (2022). Standards for Objectivity and reproducibility in high-impact developmental studies—the COVID-19 pandemic and beyond. JAMA Pediatrics.

[34] Provenzi L, Grumi S, Giorda R, Biasucci G, Bonini R, Cavallini A (2020). Measuring the outcomes of maternal COVID-19-related prenatal exposure (MOM-COPE): study protocol for a multicentric longitudinal project. BMJ Open.

[35] Beck AT, Steer RA, Carbin MG (1988). Psychometric properties of the beck depression inventory: twenty-five years of evaluation. Clin Psychol Rev.

[36] Spielberger CD, Gorsuch RL, Lushene RE, Vagg PR, Jacobs GA. State-trait anxiety inventory. Palo Alto, CA: Consulting Psychologists Press. 1970.

[37] Sica C, Ghisi M. The Italian versions of the Beck Anxiety Inventory and the Beck Depression Inventory-II: Psychometric properties and discriminant power. In Lange MA (Ed.), Leading-edge psychological tests and testing research (pp. 27–50). Nova Science Publishers.

[38] Pedrabissi L, Santinello M. Inventario per l’ansia di «Stato» e di «Tratto»: nuova versione italiana dello STAI Forma Y: manuale. Firenze: Organizzazioni Speciali. 1989;44.

[39] Montirosso R, Provenzi L, Fumagalli M, Sirgiovanni I, Giorda R, Pozzoli U (2016). Serotonin transporter gene (SLC6A4) methylation associates with neonatal intensive care unit stay and 3‐month‐old temperament in preterm infants. Child Dev.

[40] Berretta E, Guida E, Forni D, Provenzi L (2021). Glucocorticoid receptor gene (NR3C1) methylation during the first thousand days: Environmental exposures and developmental outcomes. Neurosci Biobehav Rev.

[41] Kiers HAL (1994). Simplimax: oblique rotation to an optimal target with simple structure. Psychometrika.

[42] Gallucci M. GAMLj: General analyses for linear models. [jamovi module]. Retrieved from https://gamlj.github.io/ 2019.

[43] The jamovi project, Jamovi. (Version 2.2.5.) Sidney, Australia. [Computer Software]. Retrieved from https://www.jamovi.org 2021.

[44] Sosnowski DW, Booth C, York TP, Amstadter AB, Kliewer W (2018). Maternal prenatal stress and infant DNA methylation: a systematic review. Dev Psychobiol.

[45] Stonawski V, Frey S, Golub Y, Rohleder N, Kriebel J, Goecke TW (2018). Associations of prenatal depressive symptoms with DNA methylation of HPA axis-related genes and diurnal cortisol profiles in primary school-aged children. Dev Psychopathol.

[46] DeLano K, Folger AT, Ding L, Ji H, Yolton K, Ammerman RT (2020). Associations between maternal community deprivation and infant DNA methylation of the SLC6A4 gene. Front Public Health.

[47] Dereix AE, Ledyard R, Redhunt AM, Bloomquist TR, Brennan KJ, Baccarelli AA (2021). Maternal anxiety and depression in pregnancy and DNA methylation of the NR3C1 glucocorticoid receptor gene. Epigenomics.

[48] Hompes T, Izzi B, Gellens E, Morreels M, Fieuws S, Pexsters A (2013). Investigating the influence of maternal cortisol and emotional state during pregnancy on the DNA methylation status of the glucocorticoid receptor gene (NR3C1) promoter region in cord blood. J Psychiatr Res.

[49] Palma-Gudiel H, Córdova-Palomera A, Eixarch E, Deuschle M, Fañanás L (2015). Maternal psychosocial stress during pregnancy alters the epigenetic signature of the glucocorticoid receptor gene promoter in their offspring: a meta-analysis. Epigenetics.

[50] Nazzari S, Fearon P, Rice F, Ciceri F, Molteni M, Frigerio A (2020). The biological underpinnings of perinatal depressive symptoms: a multi-systems approach. J Affect Disord.

[51] Nazzari S, Molteni M, Valtorta F, Comai S, Frigerio A (2020). Prenatal IL-6 levels and activation of the tryptophan to kynurenine pathway are associated with depressive but not anxiety symptoms across the perinatal and the post-partum period in a low-risk sample. Brain Behav Immun.

[52] Murphy VE, Smith R, Giles WB, Clifton VL (2006). Endocrine regulation of human fetal growth: the role of the mother, placenta, and fetus. Endocr Rev.

[53] Moisiadis VG, Matthews SG (2014). Glucocorticoids and fetal programming part 1: outcomes. Nat Rev Endocrinol.

[54] Dipietro JA, Irizarry RA, Costigan KA, Gurewitsch ED (2004). The psychophysiology of the maternal-fetal relationship. Psychophysiology.

[55] Turecki G, Meaney MJ (2016). Effects of the social environment and stress on glucocorticoid receptor gene methylation: a systematic review. Biol Psychiatry.

[56] McGowan PO, Sasaki A, D’Alessio AC, Dymov S, Labonté B, Szyf M (2009). Epigenetic regulation of the glucocorticoid receptor in human brain associates with childhood abuse. Nat Neurosci.

[57] Kertes DA, Kamin HS, Hughes DA, Rodney NC, Bhatt S, Mulligan CJ (2016). Prenatal maternal stress predicts methylation of genes regulating the hypothalamic-pituitary-adrenocortical system in mothers and newborns in the Democratic Republic of Congo. Child Dev.

[58] Schechter DS, Moser DA, Paoloni-Giacobino A, Stenz L, Gex-Fabry M, Aue T (2015). Methylation of NR3C1 is related to maternal PTSD, parenting stress and maternal medial prefrontal cortical activity in response to child separation among mothers with histories of violence exposure. Front Psychol.

[59] Perroud N, Rutembesa E, Paoloni-Giacobino A, Mutabaruka J, Mutesa L, Stenz L (2014). The Tutsi genocide and transgenerational transmission of maternal stress: epigenetics and biology of the HPA axis. World J Biol Psychiatry.

[60] Jung C, Ho JT, Torpy DJ, Rogers A, Doogue M, Lewis JG (2011). A longitudinal study of plasma and urinary cortisol in pregnancy and postpartum. J Clin Endocrinol Metab.

[61] Christian LM, Porter K (2014). Longitudinal changes in serum proinflammatory markers across pregnancy and postpartum: effects of maternal body mass index. Am J Managed Care.

[62] Armstrong DA, Lesseur C, Conradt E, Lester BM, Marsit CJ (2014). Global and gene-specific DNA methylation across multiple tissues in early infancy: implications for children’s health research. FASEB J.

[63] Palma-Gudiel H, Cirera F, Crispi F, Eixarch E, Fañanás L (2018). The impact of prenatal insults on the human placental epigenome: a systematic review. Neurotoxicology Teratol.

[64] Thompson TM, Sharfi D, Lee M, Yrigollen CM, Naumova OY, Grigorenko EL (2013). Comparison of whole-genome DNA methylation patterns in whole blood, saliva, and lymphoblastoid cell lines. Behav Genet.

[65] Yehuda R, Flory JD, Bierer LM, Henn-Haase C, Lehrner A, Desarnaud F (2015). Lower methylation of glucocorticoid receptor gene promoter 1F in peripheral blood of veterans with posttraumatic stress disorder. Biol Psychiatry.

[66] Schiele MA, Zwanzger P, Schwarte K, Arolt V, Baune BT, Domschke K (2021). Serotonin transporter gene promoter hypomethylation as a predictor of antidepressant treatment response in major depression: a replication study. Int J Neuropsychopharmacol.

[67] Conradt E, Lester BM, Appleton AA, Armstrong DA, Marsit CJ (2013). The roles of DNA methylation of NR3C1 and 11β-HSD2 and exposure to maternal mood disorder in utero on newborn neurobehavior. Epigenetics.

