# Supplementary material for: Maternal and infant NR3C1 and SLC6A4 epigenetic signatures of the COVID-19 pandemic lockdown: when timing matters
Source: Transl Psychiatry. 2022 Sep 16;12:386. doi: 10.1038/s41398-022-02160-0 (PMC9481531; doi:10.1038/s41398-022-02160-0)
Supplement: Supplementary file 1 — Supplementary File S1 [file 41398_2022_2160_MOESM1_ESM.docx]

**Supplementary File S1**

List of positions for the target CpG sites.

| **Gene** | **Position (hg19)** | **CpG No.** |
| --- | --- | --- |
| SLC6A4_Exon_1 | chr17:28562783-28562784 | 1 |
| SLC6A4_Exon_1 | chr17:28562786-28562787 | 2 |
| SLC6A4_Exon_1 | chr17:28562813-28562814 | 3 |
| SLC6A4_Exon_1 | chr17:28562826-28562827 | 4 |
| SLC6A4_Exon_1 | chr17:28562847-28562848 | 5 |
| SLC6A4_Exon_1 | chr17:28562849-28562850 | 6 |
| SLC6A4_Exon_1 | chr17:28562853-28562854 | 7 |
| SLC6A4_Exon_1 | chr17:28562855-28562856 | 8 |
| SLC6A4_Exon_1 | chr17:28562861-28562862 | 9 |
| SLC6A4_Exon_1 | chr17:28562863-28562864 | 10 |
| SLC6A4_Exon_1 | chr17:28562869-28562870 | 11 |
| SLC6A4_Exon_1 | chr17:28562884-28562885 | 12 |
| SLC6A4_Exon_1 | chr17:28562888-28562889 | 13 |
| Nr3c1_EXON_17-1 | chr5:142783566-142783567 | 1 |
| Nr3c1_EXON_17-1 | chr5:142783569-142783570 | 2 |
| Nr3c1_EXON_17-1 | chr5:142783584-142783585 | 3 |
| Nr3c1_EXON_17-1 | chr5:142783607-142783608 | 4 |
| Nr3c1_EXON_17-1 | chr5:142783621-142783622 | 5 |
| Nr3c1_EXON_17-1 | chr5:142783627-142783628 | 6 |
| Nr3c1_EXON_17-1 | chr5:142783637-142783638 | 7 |
| Nr3c1_EXON_17-1 | chr5:142783639-142783640 | 8 |
| Nr3c1_EXON_17-1 | chr5:142783655-142783656 | 9 |
| Nr3c1_EXON_17-1 | chr5:142783663-142783664 | 10 |
| Nr3c1_EXON_17-1 | chr5:142783678-142783679 | 11 |
| Nr3c1_EXON_17-1 | chr5:142783685-142783686 | 12 |
| Nr3c1_EXON_17-1 | chr5:142783688-142783689 | 13 |
| Nr3c1_EXON_17-1 | chr5:142783702-142783703 | 14 |
| Nr3c1_EXON_17-1 | chr5:142783712-142783713 | 15 |
| Nr3c1_EXON_17-1 | chr5:142783716-142783717 | 16 |
| Nr3c1_EXON_17-1 | chr5:142783730-142783731 | 17 |
| Nr3c1_EXON_17-1 | chr5:142783735-142783736 | 18 |
| Nr3c1_EXON_17-1 | chr5:142783742-142783743 | 19 |
| Nr3c1_EXON_17-1 | chr5:142783744-142783745 | 20 |
| Nr3c1_EXON_17-1 | chr5:142783755-142783756 | 21 |
| Nr3c1_EXON_17-1 | chr5:142783766-142783767 | 22 |
| Nr3c1_EXON_17-1 | chr5:142783768-142783769 | 23 |
| Nr3c1_EXON_17-1 | chr5:142783771-142783772 | 24 |
| Nr3c1_EXON_17-1 | chr5:142783774-142783775 | 25 |
| Nr3c1_EXON_17-1 | chr5:142783777-142783778 | 26 |
| Nr3c1_EXON_17-1 | chr5:142783780-142783781 | 27 |
| Nr3c1_EXON_17-1 | chr5:142783785-142783786 | 28 |
| Nr3c1_EXON_17-2 | chr5:142783912-142783913 | 29 |
| Nr3c1_EXON_17-2 | chr5:142783920-142783921 | 30 |
| Nr3c1_EXON_17-2 | chr5:142783927-142783928 | 31 |
| Nr3c1_EXON_17-2 | chr5:142783930-142783931 | 32 |
| Nr3c1_EXON_17-2 | chr5:142783936-142783937 | 33 |
| Nr3c1_EXON_17-2 | chr5:142783949-142783950 | 34 |
| Nr3c1_EXON_17-2 | chr5:142783959-142783960 | 35 |
| Nr3c1_EXON_17-2 | chr5:142783961-142783962 | 36 |
| Nr3c1_EXON_17-2 | chr5:142783966-142783967 | 37 |
| Nr3c1_EXON_17-2 | chr5:142783984-142783985 | 38 |
| Nr3c1_EXON_17-2 | chr5:142783997-142783998 | 39 |
| Nr3c1_EXON_17-2 | chr5:142784001-142784002 | 40 |
| Nr3c1_EXON_17-2 | chr5:142784016-142784017 | 41 |
| Nr3c1_EXON_17-2 | chr5:142784019-142784020 | 42 |
| Nr3c1_EXON_17-2 | chr5:142784022-142784023 | 43 |
| Nr3c1_EXON_17-2 | chr5:142784024-142784025 | 44 |
