# Supplementary material for: Maternal and infant NR3C1 and SLC6A4 epigenetic signatures of the COVID-19 pandemic lockdown: when timing matters
Source: Transl Psychiatry. 2022 Sep 16;12:386. doi: 10.1038/s41398-022-02160-0 (PMC9481531; doi:10.1038/s41398-022-02160-0)
Supplement: Supplementary file 2 — Supplementary File S2 [file 41398_2022_2160_MOESM2_ESM.docx]

**Supplementary File S2**

The following tables report the results of the principal component analysis conducted on maternal and infants’ *NR3C1* and SLC6A4 methylation at specific CpG sites.

*[A] SLC6A4 (mothers)*

| Component Loadings | | | | | |
| --- | --- | --- | --- | --- | --- |
|  | | **Component** | |  | |
|  | | **1** | | **Uniqueness** | |
| slc6a4_1 |  | 0.798 |  | 0.364 |  |
| slc6a4_2 |  | 0.670 |  | 0.551 |  |
| slc6a4_3 |  | 0.598 |  | 0.643 |  |
| slc6a4_4 |  | 0.726 |  | 0.473 |  |
| slc6a4_5 |  | 0.828 |  | 0.314 |  |
| slc6a4_6 |  | 0.740 |  | 0.453 |  |
| slc6a4_7 |  | 0.566 |  | 0.680 |  |
| slc6a4_8 |  |  |  | 0.885 |  |
| slc6a4_9 |  | 0.842 |  | 0.291 |  |
| slc6a4_10 |  | 0.726 |  | 0.473 |  |
| slc6a4_11 |  | 0.759 |  | 0.425 |  |
| slc6a4_12 |  | 0.844 |  | 0.288 |  |
| slc6a4_13 |  | 0.682 |  | 0.535 |  |
| Note. 'simplimax' rotation was used | | | | | |
|  | | | | | |

*[B] SLC6A4 (infants)*

| Component Loadings | | | | | | | | | | | | | |
| --- | --- | --- | --- | --- | --- | --- | --- | --- | --- | --- | --- | --- | --- |
|  | | **Component** | | | | | | | | | |  | |
|  | | **1** | | **2** | | **3** | | **4** | | **5** | | **Uniqueness** | |
| slc6a4_1 |  | 0.697 |  |  |  |  |  |  |  |  |  | 0.443 |  |
| slc6a4_2 |  | 0.439 |  | -0.752 |  |  |  |  |  |  |  | 0.125 |  |
| slc6a4_3 |  | 0.518 |  |  |  |  |  | 0.469 |  | 0.509 |  | 0.302 |  |
| slc6a4_4 |  |  |  |  |  |  |  | 0.876 |  |  |  | 0.157 |  |
| slc6a4_5 |  | 0.564 |  |  |  |  |  |  |  |  |  | 0.501 |  |
| slc6a4_6 |  |  |  | -0.749 |  |  |  |  |  |  |  | 0.112 |  |
| slc6a4_7 |  | 0.438 |  |  |  | 0.459 |  |  |  | 0.475 |  | 0.298 |  |
| slc6a4_8 |  | 0.567 |  |  |  |  |  |  |  |  |  | 0.585 |  |
| slc6a4_9 |  | 0.674 |  |  |  |  |  |  |  |  |  | 0.491 |  |
| slc6a4_10 |  | 0.620 |  |  |  | -0.404 |  |  |  |  |  | 0.404 |  |
| slc6a4_11 |  | 0.499 |  |  |  |  |  |  |  | -0.523 |  | 0.411 |  |
| slc6a4_12 |  | 0.777 |  |  |  |  |  |  |  |  |  | 0.321 |  |
| slc6a4_13 |  |  |  |  |  | 0.512 |  |  |  | -0.514 |  | 0.232 |  |
| Note. 'simplimax' rotation was used | | | | | | | | | | | | | |
|  | | | | | | | | | | | | | |

*[C] NR3C1 (mothers)*

| Component Loadings | | | | | | | | | | | | | | | | | | | | | | | | | | | | | | | | | |
| --- | --- | --- | --- | --- | --- | --- | --- | --- | --- | --- | --- | --- | --- | --- | --- | --- | --- | --- | --- | --- | --- | --- | --- | --- | --- | --- | --- | --- | --- | --- | --- | --- | --- |
|  | | **Component** | | | | | | | | | | | | | | | | | | | | | | | | | | | | | |  | |
|  | | **1** | | **2** | | **3** | | **4** | | **5** | | **6** | | **7** | | **8** | | **9** | | **10** | | **11** | | **12** | | **13** | | **14** | | **15** | | **Uniqueness** | |
| nr3c1_1 |  |  |  |  |  |  |  |  |  |  |  |  |  |  |  |  |  |  |  | 0.523 |  |  |  |  |  |  |  |  |  |  |  | 0.588 |  |
| nr3c1_2 |  |  |  |  |  |  |  |  |  |  |  |  |  |  |  |  |  |  |  |  |  |  |  |  |  |  |  |  |  |  |  | 0.452 |  |
| nr3c1_3 |  |  |  | 0.550 |  |  |  |  |  |  |  |  |  |  |  |  |  |  |  |  |  |  |  |  |  |  |  |  |  |  |  | 0.342 |  |
| nr3c1_4 |  |  |  |  |  |  |  |  |  |  |  |  |  |  |  |  |  |  |  |  |  | 0.405 |  |  |  |  |  |  |  |  |  | 0.365 |  |
| nr3c1_5 |  |  |  |  |  |  |  | 0.560 |  |  |  |  |  |  |  |  |  |  |  |  |  |  |  |  |  |  |  |  |  |  |  | 0.355 |  |
| nr3c1_6 |  |  |  |  |  |  |  |  |  |  |  |  |  |  |  |  |  |  |  |  |  |  |  |  |  |  |  |  |  |  |  | 0.464 |  |
| nr3c1_7 |  |  |  |  |  |  |  |  |  |  |  |  |  |  |  |  |  |  |  |  |  |  |  |  |  |  |  |  |  |  |  | 0.382 |  |
| nr3c1_8 |  |  |  |  |  |  |  |  |  |  |  |  |  |  |  |  |  |  |  |  |  |  |  |  |  |  |  |  |  |  |  | 0.410 |  |
| nr3c1_9 |  |  |  |  |  |  |  |  |  |  |  |  |  |  |  |  |  |  |  |  |  |  |  |  |  |  |  |  |  | 0.405 |  | 0.336 |  |
| nr3c1_10 |  |  |  |  |  |  |  |  |  |  |  |  |  |  |  |  |  | 0.436 |  |  |  |  |  | 0.404 |  |  |  |  |  |  |  | 0.271 |  |
| nr3c1_11 |  |  |  |  |  |  |  |  |  |  |  |  |  |  |  |  |  |  |  |  |  |  |  |  |  |  |  |  |  |  |  | 0.587 |  |
| nr3c1_12 |  |  |  |  |  |  |  |  |  |  |  |  |  |  |  |  |  |  |  |  |  |  |  |  |  |  |  |  |  |  |  | 0.342 |  |
| nr3c1_13 |  | 0.451 |  |  |  |  |  |  |  |  |  |  |  |  |  |  |  |  |  |  |  |  |  |  |  |  |  |  |  |  |  | 0.368 |  |
| nr3c1_14 |  |  |  | 0.434 |  |  |  |  |  |  |  |  |  |  |  |  |  |  |  |  |  |  |  |  |  |  |  |  |  |  |  | 0.445 |  |
| nr3c1_15 |  |  |  |  |  |  |  |  |  |  |  |  |  |  |  |  |  |  |  |  |  |  |  |  |  |  |  |  |  |  |  | 0.474 |  |
| nr3c1_16 |  |  |  |  |  |  |  |  |  |  |  |  |  |  |  |  |  |  |  |  |  |  |  |  |  |  |  |  |  |  |  | 0.329 |  |
| nr3c1_17 |  | 0.422 |  |  |  | 0.406 |  |  |  |  |  |  |  |  |  |  |  |  |  |  |  |  |  |  |  |  |  |  |  |  |  | 0.491 |  |
| nr3c1_18 |  |  |  |  |  |  |  | 0.570 |  |  |  |  |  |  |  |  |  |  |  |  |  |  |  |  |  |  |  |  |  |  |  | 0.328 |  |
| nr3c1_19 |  | 0.524 |  |  |  |  |  |  |  |  |  |  |  |  |  |  |  |  |  |  |  |  |  |  |  |  |  |  |  |  |  | 0.369 |  |
| nr3c1_20 |  |  |  |  |  |  |  |  |  | -0.403 |  |  |  |  |  |  |  |  |  |  |  |  |  |  |  |  |  |  |  |  |  | 0.408 |  |
| nr3c1_21 |  |  |  | 0.575 |  |  |  |  |  |  |  |  |  |  |  |  |  |  |  |  |  |  |  |  |  |  |  |  |  |  |  | 0.328 |  |
| nr3c1_22 |  | 0.402 |  |  |  |  |  |  |  |  |  |  |  |  |  |  |  |  |  |  |  |  |  |  |  |  |  |  |  |  |  | 0.392 |  |
| nr3c1_23 |  |  |  |  |  |  |  |  |  |  |  |  |  |  |  |  |  |  |  |  |  |  |  |  |  |  |  |  |  |  |  | 0.377 |  |
| nr3c1_24 |  |  |  |  |  |  |  |  |  |  |  |  |  |  |  |  |  |  |  |  |  |  |  |  |  | 0.596 |  |  |  |  |  | 0.368 |  |
| nr3c1_25 |  |  |  |  |  | 0.403 |  |  |  |  |  |  |  |  |  |  |  |  |  |  |  |  |  |  |  |  |  |  |  |  |  | 0.482 |  |
| nr3c1_26 |  |  |  |  |  |  |  |  |  |  |  |  |  |  |  |  |  |  |  |  |  |  |  |  |  |  |  |  |  |  |  | 0.430 |  |
| nr3c1_27 |  |  |  |  |  |  |  |  |  |  |  |  |  |  |  |  |  |  |  |  |  |  |  |  |  |  |  |  |  |  |  | 0.418 |  |
| nr3c1_28 |  | 0.422 |  |  |  |  |  |  |  |  |  |  |  |  |  |  |  |  |  |  |  |  |  |  |  |  |  |  |  |  |  | 0.421 |  |
| nr3c1_1 |  | 0.426 |  |  |  |  |  |  |  |  |  | 0.412 |  |  |  |  |  |  |  |  |  |  |  |  |  |  |  |  |  |  |  | 0.408 |  |
| nr3c1_2 |  | 0.410 |  |  |  |  |  |  |  |  |  |  |  |  |  |  |  |  |  |  |  |  |  |  |  |  |  |  |  |  |  | 0.323 |  |
| nr3c1_3 |  | 0.552 |  |  |  |  |  |  |  |  |  |  |  |  |  |  |  |  |  |  |  |  |  |  |  |  |  |  |  |  |  | 0.398 |  |
| nr3c1_4 |  | 0.535 |  |  |  |  |  |  |  |  |  |  |  |  |  |  |  |  |  |  |  |  |  |  |  |  |  |  |  |  |  | 0.338 |  |
| nr3c1_5 |  |  |  |  |  |  |  |  |  | -0.454 |  |  |  |  |  |  |  |  |  |  |  |  |  |  |  |  |  |  |  |  |  | 0.309 |  |
| nr3c1_6 |  | 0.581 |  |  |  |  |  |  |  |  |  |  |  |  |  |  |  |  |  |  |  |  |  |  |  |  |  |  |  |  |  | 0.446 |  |
| nr3c1_7 |  | 0.535 |  |  |  |  |  |  |  |  |  |  |  |  |  |  |  |  |  |  |  |  |  |  |  |  |  |  |  |  |  | 0.318 |  |
| nr3c1_8 |  |  |  |  |  | -0.417 |  |  |  |  |  |  |  |  |  |  |  |  |  |  |  |  |  |  |  |  |  |  |  |  |  | 0.317 |  |
| nr3c1_9 |  | 0.503 |  |  |  |  |  |  |  |  |  |  |  |  |  |  |  |  |  |  |  |  |  |  |  |  |  |  |  |  |  | 0.353 |  |
| nr3c1_10 |  | 0.505 |  |  |  |  |  |  |  |  |  |  |  |  |  |  |  |  |  |  |  |  |  |  |  |  |  |  |  |  |  | 0.299 |  |
| nr3c1_11 |  |  |  | 0.730 |  |  |  |  |  |  |  |  |  |  |  |  |  |  |  |  |  |  |  |  |  |  |  |  |  |  |  | 0.231 |  |
| nr3c1_12 |  |  |  | 0.680 |  |  |  |  |  |  |  |  |  |  |  |  |  |  |  |  |  |  |  |  |  |  |  |  |  |  |  | 0.230 |  |
| nr3c1_13 |  |  |  |  |  |  |  |  |  | 0.459 |  |  |  |  |  |  |  |  |  |  |  |  |  |  |  |  |  |  |  |  |  | 0.338 |  |
| nr3c1_14 |  | 0.549 |  |  |  |  |  |  |  |  |  |  |  |  |  |  |  |  |  |  |  |  |  |  |  |  |  |  |  |  |  | 0.380 |  |
| nr3c1_15 |  | 0.459 |  |  |  |  |  |  |  |  |  |  |  | 0.420 |  |  |  |  |  |  |  |  |  |  |  |  |  |  |  |  |  | 0.375 |  |
| nr3c1_16 |  |  |  | 0.479 |  |  |  |  |  |  |  |  |  |  |  |  |  |  |  |  |  |  |  |  |  |  |  |  |  |  |  | 0.330 |  |
| Note. 'simplimax' rotation was used | | | | | | | | | | | | | | | | | | | | | | | | | | | | | | | | | |
|  | | | | | | | | | | | | | | | | | | | | | | | | | | | | | | | | | |

*[D] NR3C1 (infants)*

| Component Loadings | | | | | | | | | | | | | | | | | | | | | | | | | | | | | | | | | | | |
| --- | --- | --- | --- | --- | --- | --- | --- | --- | --- | --- | --- | --- | --- | --- | --- | --- | --- | --- | --- | --- | --- | --- | --- | --- | --- | --- | --- | --- | --- | --- | --- | --- | --- | --- | --- |
|  | | **Component** | | | | | | | | | | | | | | | | | | | | | | | | | | | | | | | |  | |
|  | | **1** | | **2** | | **3** | | **4** | | **5** | | **6** | | **7** | | **8** | | **9** | | **10** | | **11** | | **12** | | **13** | | **14** | | **15** | | **16** | | **Uniqueness** | |
| nr3c1_1 |  |  |  |  |  |  |  |  |  |  |  |  |  |  |  |  |  |  |  |  |  |  |  |  |  |  |  |  |  |  |  |  |  | 0.468 |  |
| nr3c1_2 |  |  |  |  |  |  |  |  |  |  |  |  |  |  |  | 0.431 |  |  |  |  |  |  |  |  |  |  |  |  |  |  |  |  |  | 0.474 |  |
| nr3c1_3 |  |  |  |  |  |  |  |  |  |  |  |  |  |  |  |  |  |  |  |  |  |  |  |  |  |  |  |  |  |  |  |  |  | 0.465 |  |
| nr3c1_4 |  |  |  |  |  |  |  |  |  |  |  |  |  |  |  |  |  |  |  |  |  |  |  | 0.408 |  |  |  |  |  |  |  |  |  | 0.408 |  |
| nr3c1_5 |  | 0.432 |  |  |  |  |  |  |  |  |  |  |  |  |  |  |  |  |  |  |  |  |  |  |  |  |  |  |  |  |  |  |  | 0.467 |  |
| nr3c1_6 |  |  |  |  |  |  |  |  |  |  |  |  |  |  |  |  |  |  |  |  |  |  |  |  |  |  |  |  |  |  |  |  |  | 0.451 |  |
| nr3c1_7 |  |  |  |  |  |  |  |  |  |  |  |  |  |  |  |  |  |  |  |  |  |  |  |  |  |  |  |  |  |  |  |  |  | 0.431 |  |
| nr3c1_8 |  |  |  |  |  |  |  |  |  |  |  |  |  |  |  |  |  |  |  |  |  |  |  |  |  |  |  |  |  |  |  |  |  | 0.446 |  |
| nr3c1_9 |  |  |  | 0.779 |  |  |  |  |  |  |  |  |  |  |  |  |  |  |  |  |  |  |  |  |  |  |  |  |  |  |  |  |  | 0.282 |  |
| nr3c1_10 |  |  |  |  |  |  |  |  |  |  |  |  |  | 0.533 |  |  |  |  |  |  |  |  |  |  |  |  |  |  |  |  |  |  |  | 0.169 |  |
| nr3c1_11 |  |  |  |  |  |  |  |  |  |  |  |  |  |  |  |  |  |  |  |  |  |  |  |  |  |  |  |  |  |  |  |  |  | 0.429 |  |
| nr3c1_12 |  |  |  |  |  |  |  |  |  |  |  |  |  |  |  |  |  |  |  |  |  |  |  |  |  |  |  |  |  |  |  |  |  | 0.529 |  |
| nr3c1_13 |  | 0.427 |  |  |  | 0.425 |  |  |  |  |  |  |  |  |  |  |  |  |  |  |  |  |  |  |  |  |  |  |  |  |  |  |  | 0.456 |  |
| nr3c1_14 |  |  |  |  |  |  |  |  |  |  |  |  |  |  |  |  |  |  |  |  |  |  |  |  |  |  |  | 0.411 |  |  |  |  |  | 0.347 |  |
| nr3c1_15 |  |  |  |  |  |  |  |  |  |  |  |  |  |  |  |  |  |  |  |  |  |  |  |  |  |  |  |  |  |  |  |  |  | 0.431 |  |
| nr3c1_16 |  |  |  |  |  |  |  |  |  |  |  | -0.485 |  |  |  |  |  |  |  |  |  |  |  |  |  |  |  |  |  |  |  |  |  | 0.400 |  |
| nr3c1_17 |  |  |  |  |  |  |  |  |  |  |  |  |  |  |  |  |  |  |  |  |  |  |  |  |  |  |  |  |  |  |  |  |  | 0.358 |  |
| nr3c1_18 |  |  |  |  |  |  |  |  |  |  |  |  |  |  |  |  |  |  |  |  |  |  |  |  |  |  |  |  |  | -0.417 |  |  |  | 0.392 |  |
| nr3c1_19 |  |  |  |  |  |  |  |  |  |  |  |  |  |  |  |  |  |  |  |  |  |  |  |  |  | -0.443 |  |  |  |  |  |  |  | 0.284 |  |
| nr3c1_20 |  |  |  |  |  |  |  |  |  |  |  |  |  |  |  |  |  |  |  |  |  |  |  |  |  |  |  |  |  |  |  |  |  | 0.414 |  |
| nr3c1_21 |  |  |  | 0.752 |  |  |  |  |  |  |  |  |  |  |  |  |  |  |  |  |  |  |  |  |  |  |  |  |  |  |  |  |  | 0.302 |  |
| nr3c1_22 |  |  |  |  |  |  |  |  |  |  |  |  |  |  |  |  |  |  |  |  |  |  |  |  |  |  |  |  |  | 0.482 |  |  |  | 0.352 |  |
| nr3c1_23 |  |  |  | 0.652 |  |  |  |  |  |  |  |  |  |  |  |  |  |  |  |  |  |  |  |  |  |  |  |  |  |  |  |  |  | 0.325 |  |
| nr3c1_24 |  |  |  |  |  |  |  |  |  |  |  |  |  |  |  |  |  |  |  |  |  |  |  |  |  |  |  |  |  |  |  |  |  | 0.392 |  |
| nr3c1_25 |  |  |  |  |  |  |  |  |  |  |  |  |  |  |  |  |  |  |  |  |  |  |  |  |  |  |  |  |  |  |  |  |  | 0.369 |  |
| nr3c1_26 |  |  |  |  |  |  |  |  |  |  |  |  |  |  |  |  |  |  |  |  |  |  |  |  |  |  |  |  |  |  |  |  |  | 0.306 |  |
| nr3c1_27 |  | 0.490 |  |  |  |  |  |  |  |  |  |  |  |  |  |  |  | -0.438 |  |  |  |  |  |  |  |  |  |  |  |  |  |  |  | 0.332 |  |
| nr3c1_28 |  | 0.514 |  |  |  |  |  |  |  |  |  |  |  |  |  |  |  |  |  |  |  | 0.495 |  |  |  |  |  |  |  |  |  |  |  | 0.370 |  |
| nr3c1_1 |  | 0.570 |  |  |  |  |  |  |  |  |  |  |  |  |  |  |  |  |  |  |  |  |  |  |  |  |  |  |  |  |  |  |  | 0.464 |  |
| nr3c1_2 |  | 0.567 |  |  |  |  |  |  |  |  |  |  |  |  |  |  |  |  |  |  |  |  |  |  |  |  |  |  |  |  |  |  |  | 0.303 |  |
| nr3c1_3 |  | 0.513 |  |  |  |  |  |  |  |  |  |  |  |  |  |  |  |  |  |  |  |  |  |  |  |  |  |  |  |  |  |  |  | 0.338 |  |
| nr3c1_4 |  |  |  |  |  | -0.405 |  |  |  |  |  |  |  |  |  |  |  |  |  |  |  |  |  |  |  |  |  |  |  |  |  |  |  | 0.195 |  |
| nr3c1_5 |  |  |  |  |  |  |  |  |  |  |  |  |  |  |  |  |  |  |  |  |  |  |  |  |  |  |  |  |  |  |  |  |  | 0.473 |  |
| nr3c1_6 |  | 0.631 |  |  |  |  |  |  |  |  |  |  |  |  |  |  |  |  |  |  |  |  |  |  |  |  |  |  |  |  |  |  |  | 0.383 |  |
| nr3c1_7 |  | 0.481 |  |  |  |  |  |  |  |  |  |  |  |  |  |  |  |  |  |  |  |  |  |  |  |  |  |  |  |  |  |  |  | 0.242 |  |
| nr3c1_8 |  | 0.493 |  |  |  |  |  |  |  |  |  |  |  |  |  |  |  |  |  |  |  |  |  |  |  |  |  |  |  |  |  |  |  | 0.407 |  |
| nr3c1_9 |  |  |  |  |  | 0.494 |  |  |  |  |  |  |  |  |  |  |  |  |  |  |  |  |  |  |  |  |  |  |  |  |  |  |  | 0.267 |  |
| nr3c1_10 |  | 0.486 |  |  |  |  |  |  |  |  |  |  |  |  |  |  |  |  |  |  |  |  |  |  |  |  |  |  |  |  |  |  |  | 0.426 |  |
| nr3c1_11 |  |  |  |  |  |  |  |  |  |  |  |  |  |  |  |  |  |  |  |  |  |  |  |  |  |  |  |  |  |  |  |  |  | 0.320 |  |
| nr3c1_12 |  | 0.488 |  |  |  |  |  | -0.422 |  |  |  |  |  |  |  |  |  |  |  |  |  |  |  |  |  |  |  |  |  |  |  |  |  | 0.259 |  |
| nr3c1_13 |  | 0.518 |  |  |  |  |  |  |  |  |  |  |  |  |  |  |  |  |  |  |  |  |  |  |  |  |  |  |  |  |  |  |  | 0.302 |  |
| nr3c1_14 |  | 0.460 |  | 0.503 |  |  |  |  |  |  |  |  |  |  |  |  |  |  |  |  |  |  |  |  |  |  |  |  |  |  |  |  |  | 0.334 |  |
| nr3c1_15 |  | 0.476 |  |  |  |  |  |  |  |  |  |  |  |  |  | 0.436 |  |  |  |  |  |  |  |  |  |  |  |  |  |  |  |  |  | 0.274 |  |
| nr3c1_16 |  | 0.413 |  |  |  |  |  | 0.432 |  |  |  |  |  |  |  |  |  |  |  |  |  |  |  |  |  |  |  |  |  |  |  |  |  | 0.439 |  |
| Note. 'simplimax' rotation was used | | | | | | | | | | | | | | | | | | | | | | | | | | | | | | | | | | | |
|  | | | | | | | | | | | | | | | | | | | | | | | | | | | | | | | | | | | |
