# Supplementary material for: Maternal and infant NR3C1 and SLC6A4 epigenetic signatures of the COVID-19 pandemic lockdown: when timing matters
Source: Transl Psychiatry. 2022 Sep 16;12:386. doi: 10.1038/s41398-022-02160-0 (PMC9481531; doi:10.1038/s41398-022-02160-0)
Supplement: Supplementary file 3 — Supplementary File S3 [file 41398_2022_2160_MOESM3_ESM.docx]

**Supplementary File S3**

The following tables report the results of the ANCOVAs conducted on methylation PCs checking for the effect of maternal age as covariate.

| ANCOVA - M-SLC6A4 | | | | | | | | | | | |
| --- | --- | --- | --- | --- | --- | --- | --- | --- | --- | --- | --- |
|  |  |  |  |  |  |  |  |  |  |  |  |
|  | | **Sum of Squares** | | **df** | | **Mean Square** | | **F** | | **p** | |
| trim |  | 38.77 |  | 2 |  | 19.384 |  | 22.22 |  | < .001 |  |
| m_age |  | 3.04 |  | 1 |  | 3.038 |  | 3.48 |  | 0.063 |  |
| Residuals |  | 242.47 |  | 278 |  | 0.872 |  |  |  |  |  |
|  | | | | | | | | | | | |

| ANCOVA - I-SLC6A4 | | | | | | | | | | | |
| --- | --- | --- | --- | --- | --- | --- | --- | --- | --- | --- | --- |
|  |  |  |  |  |  |  |  |  |  |  |  |
|  | | **Sum of Squares** | | **df** | | **Mean Square** | | **F** | | **p** | |
| trim |  | 19.01 |  | 2 |  | 9.503 |  | 10.3 |  | < .001 |  |
| m_age |  | 9.63 |  | 1 |  | 9.631 |  | 10.4 |  | 0.001 |  |
| Residuals |  | 256.56 |  | 278 |  | 0.923 |  |  |  |  |  |
|  | | | | | | | | | | | |

| ANCOVA - M-NR3C1 | | | | | | | | | | | |
| --- | --- | --- | --- | --- | --- | --- | --- | --- | --- | --- | --- |
|  |  |  |  |  |  |  |  |  |  |  |  |
|  | | **Sum of Squares** | | **df** | | **Mean Square** | | **F** | | **p** | |
| trim |  | 76.4 |  | 2 |  | 38.195 |  | 52.7 |  | < .001 |  |
| m_age |  | 10.4 |  | 1 |  | 10.432 |  | 14.4 |  | < .001 |  |
| Residuals |  | 199.2 |  | 275 |  | 0.725 |  |  |  |  |  |
|  | | | | | | | | | | | |

| ANCOVA - I-NR3C1 | | | | | | | | | | | |
| --- | --- | --- | --- | --- | --- | --- | --- | --- | --- | --- | --- |
|  |  |  |  |  |  |  |  |  |  |  |  |
|  | | **Sum of Squares** | | **df** | | **Mean Square** | | **F** | | **p** | |
| trim |  | 44.5 |  | 2 |  | 22.237 |  | 26.8 |  | < .001 |  |
| m_age |  | 12.3 |  | 1 |  | 12.283 |  | 14.8 |  | < .001 |  |
| Residuals |  | 231.0 |  | 278 |  | 0.831 |  |  |  |  |  |
|  | | | | | | | | | | | |
